# Standardized 3D test object for multi-camera calibration during animal pose capture

**DOI:** 10.1117/1.NPh.10.4.046602

**Published:** 2023-11-07

**Authors:** Hao Hu, Roark Zhang, Tony Fong, Helge Rhodin, Timothy H. Murphy

**Affiliations:** a University of British Columbia, Department of Psychiatry, Kinsmen Laboratory of Neurological Research, Vancouver, British Columbia, Canada; b University of British Columbia, Djavad Mowafaghian Centre for Brain Health, Vancouver, British Columbia, Canada; c University of British Columbia, Department of Computer Science, Vancouver, British Columbia, Canada

**Keywords:** camera calibration, error analysis, three-dimensional animal behavior tracking

## Abstract

Accurate capture of animal behavior and posture requires the use of multiple cameras to reconstruct three-dimensional (3D) representations. Typically, a paper ChArUco (or checker) board works well for correcting distortion and calibrating for 3D reconstruction in stereo vision. However, measuring the error in two-dimensional (2D) is also prone to bias related to the placement of the 2D board in 3D. We proposed a procedure as a visual way of validating camera placement, and it also can provide some guidance about the positioning of cameras and potential advantages of using multiple cameras. We propose the use of a 3D printable test object for validating multi-camera surround-view calibration in small animal video capture arenas. The proposed 3D printed object has no bias to a particular dimension and is designed to minimize occlusions. The use of the calibrated test object provided an estimate of 3D reconstruction accuracy. The approach reveals that for complex specimens such as mice, some view angles will be more important for accurate capture of keypoints. Our method ensures accurate 3D camera calibration for surround image capture of laboratory mice and other specimens.

## 1 Introduction

Accurate multi-camera motion capture can provide critical insights for a wide variety of biomedical inquiries, including behavior, zoology, kinesiology, and neuroscience research.^1^^–^^7^ More specifically, the capture of motion data is essential for behavioral quantification and potential linkages with neural activity.^3^^,^^7^^,^^8^ However, accurate capture of motion may be challenging due to calibration errors and other factors. With animals, the challenges are even greater due to the general lack of training data and ground truth body plan information.^5^ Another potential issue is marker placement: most existing high-accuracy motion capture systems rely on the installation of invasive markers, which may affect natural movements and behaviors.^5^ Furthermore, three-dimensional (3D) accuracy and precision remain difficult to obtain in the context of keypoint detection using arbitrary camera setups (non-defined angles). Despite these challenges, it is imperative for biomedical motion capture systems to provide accurate, insightful data in the absence of well-defined markers.^9^

Errors in 3D markerless tracking based on a multi-camera tracking system generally can be grouped into three categories: calibration error, matching error [two-dimensional (2D) keypoints detection or pairing error], and reconstruction error.^10^ Most studies only focus on the aggregate error often termed the reprojection error in 2D images. However, errors in 2D might be misleading, prone to bias and hard to use as guidelines when assessing an animal tracking arena. A caveat is that this study was unable to encompass the entire categories of error. For example, occlusion is a major contributor to error in motion capture and can cause inaccurate or failed 2D keypoint detection. Occlusion errors will not only need to be specific to each species/body-plan examined but also to each experiment (global versus local motion, type of activities). Given this complexity, it is beyond the scope of this study to examine errors caused by occlusion (when less camera views are available).

In order to measure the performance of biomedical motion capture systems, we introduce 3D test specimens which can be used as a metric for the lower bound of the 3D-positioning error. Such easy-to-use specimens provide a visual check on 3D tracking efficacy and guidance for the number of cameras to use and their positioning. Here, we describe a method to evaluate the accuracy of 3D markerless motion capture and reconstruction data for camera setups. Our pipeline is built on existing markerless motion capture algorithms and provides a robust method to verify the accuracy and precision of any multi-camera tracking system.

## 2 Methods

### 2.1 3D Video Acquisition Arena

**Fig. 1:**
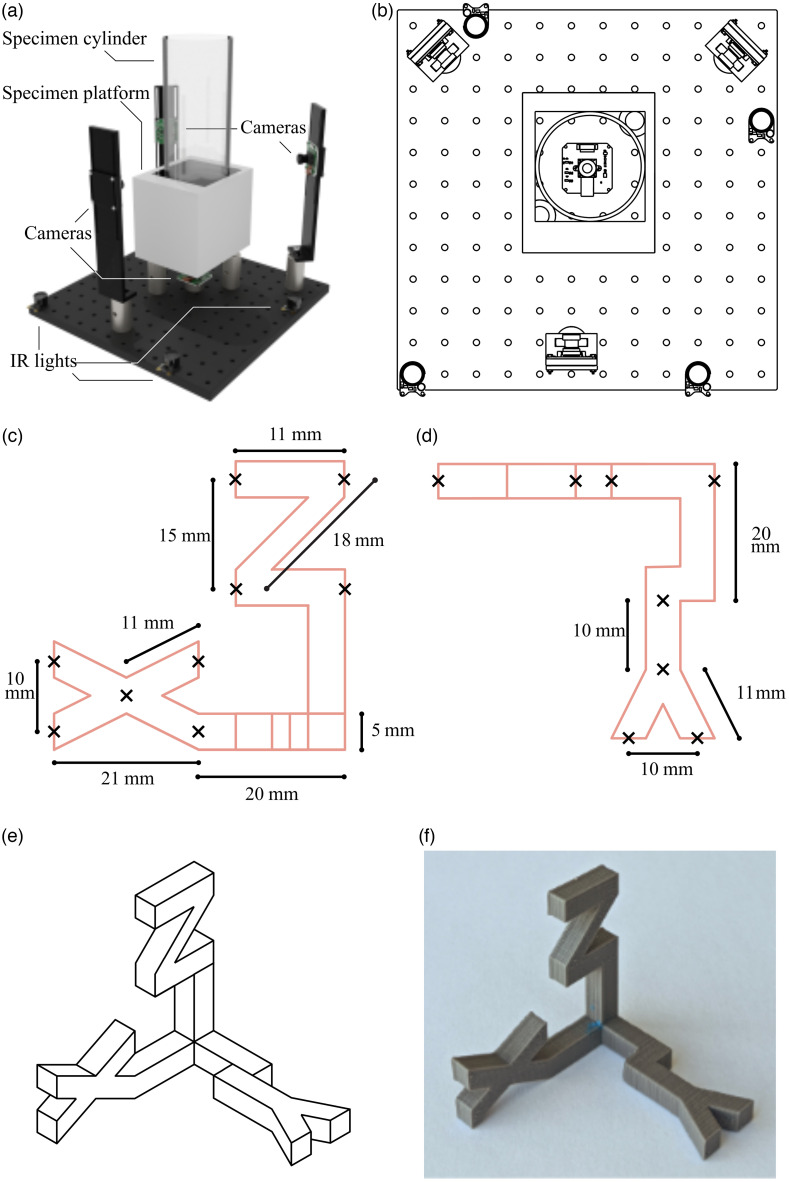
Specifications of the mouse imaging arena and 3D test object. (a) Render of the 3D small animal capture arena. (b) Schematic top-view illustration of the image capture arena. (c) Top-view schematic of 3D-printed test object annotated with distances and keypoint positions. (d) Side-view schematic of 3D-printed test object annotated with distances and keypoint positions. (e) 3D appearance of the test object. (f) Image of the 3D-printed test object.

To reconstruct the animal behavior in 3D space and validate the performance of 3D tracking systems, we built a 3D video acquisition arena and validated its performance using a 3D printed test object and real mouse data. The 3D video acquisition arena for small animals is constructed using custom 3D printed parts and an 89 mm diameter plexiglass cylinder. For image acquisition, a Quadrascopic camera solution (Arducam; SKU: B0267) consisting of four cameras synchronized at the hardware level was used. Each camera had its IR cut filter removed (by the vendor) and uses a global shutter monochrome OV9281 sensor with 1280×800 active pixels and an effective focal length of 2.4 mm. A similar 4 Raspberry Pi Picam camera rig was also used for image acquisition with 1296×972 active pixels. Three cameras were placed at 120-deg angles at a horizontal elevation around the subject. The fourth camera was placed below the subject with imaging performed through a transparent plexiglass floor. The imaging setup is shown in Fig. 1 and is available as a 3D model (see OSF repository^19^). To match most behavioral assessments, an additional larger arena was also constructed using a 320 mm diameter plexiglass plate with cameras moved 340 mm from the center.

Both the 3D test object and freely moving mouse were recorded with the same 3D video acquisition arena. These videos were analyzed using Anipose,^11^ which is an open-source, markerless 3D tracking system based on multi-camera recordings. It consists of modules for camera calibration, 2D keypoints detection (using DeepLabCut),^12^ 2D detection refinement, and 3D reconstruction. The intrinsic and extrinsic parameters for cameras were determined using video of a ChArUco (or checker) board and used for calibration.^13^^,^^14^ The board was positioned nearly upright at the center of the capture volume, ensuring that it was visible to at least two cameras and slowly rotated along the coronal axis (the bottom edge of the board) to face the bottom camera. This procedure was repeated for every camera. Different DeepLabCut models were trained for test object tracking and mouse tracking (Table 1). Then, the 3D reconstruction was performed using Anipose.

**Table 1:** Comprehensive assessment of DeepLabCut performance.

			Model type	Training data size (units: frame)	Training error (units: pixel)	Test error (units: pixel)
Test object	89 mm arena		ResNet-101	120	2.04	13.32
	320 mm arena		ResNet-101	120	2.46	8.66
Mice	89 mm arena	Side views	ResNet-101	1120	6.43	20.54
		Bottom view	ResNet-101	1467	5.2	15.52
	320 mm arena	Side views	ResNet-101	1350	8.82	10.51
		Bottom view	ResNet-101	400	2.93	5.5

**Fig. 2:**
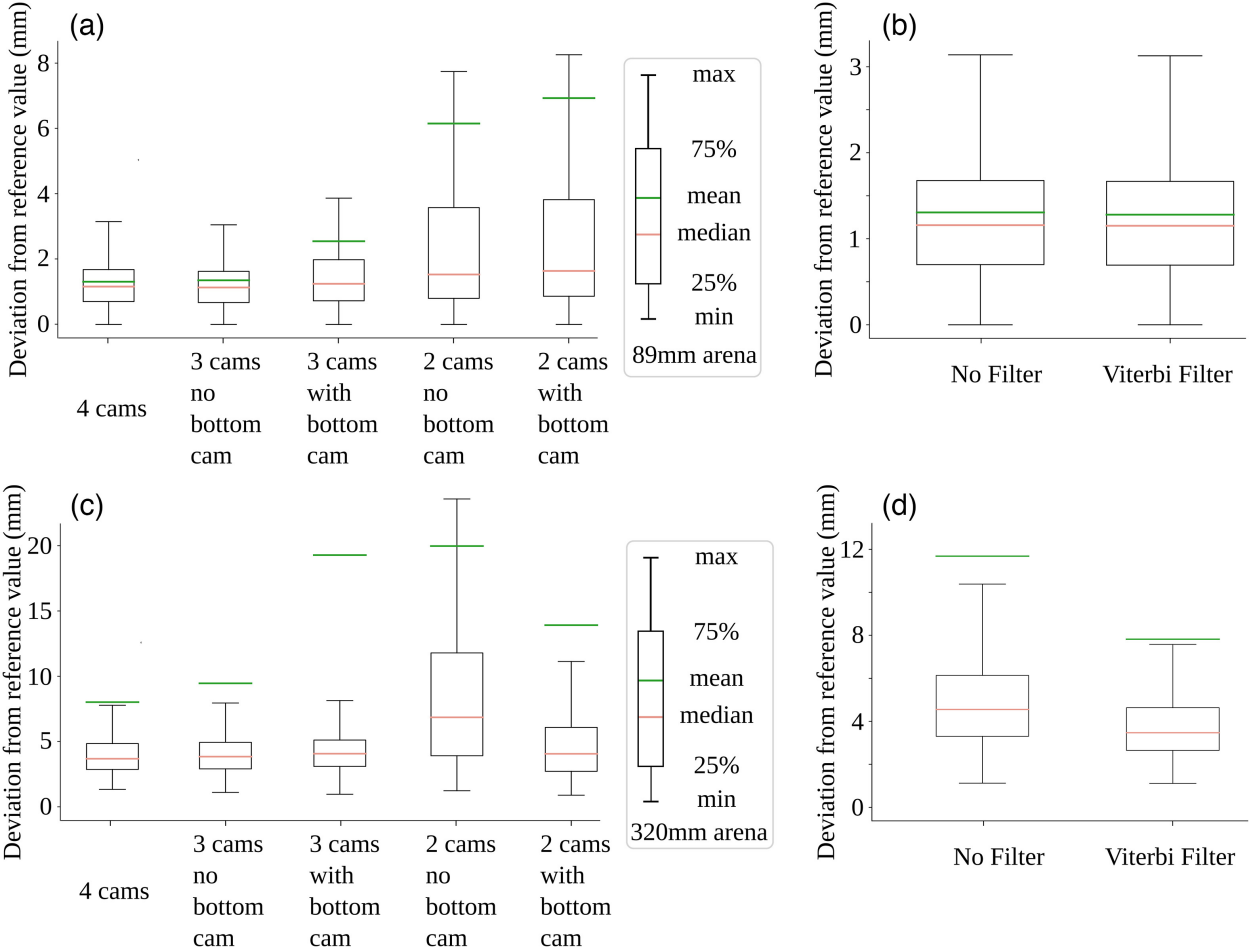
Camera configuration and software filtering effects on 3D positioning error for known test objects in small and large arenas. (a) Boxplot of error for various camera configurations in the 89 mm arena, in which the box edges represent Q1 (25th% quartiles) and Q3 (75th% quartiles), and the whiskers represent minimum (Q0 = Q1 − 1.5 IQR) and maximum (Q4 = Q3 + 1.5 IQR) where IQR refers to interquartile range Q3 to Q1. Outliers are not shown when error is greater than Q4 or less than Q0. For each configuration, all symmetrical configurations were combined into one category. No filtering was active in any configuration. (b) Boxplot of error comparing the use of a Viterbi filter. Both trials used the same four-camera source videos in an 89 mm arena. (c) Boxplot of error for various camera configurations in the 320 mm arena. (d) Boxplot of error comparing the use of a Viterbi filter. Both trials used the same four-camera source videos in a 320 mm arena.

**Fig. 3:**
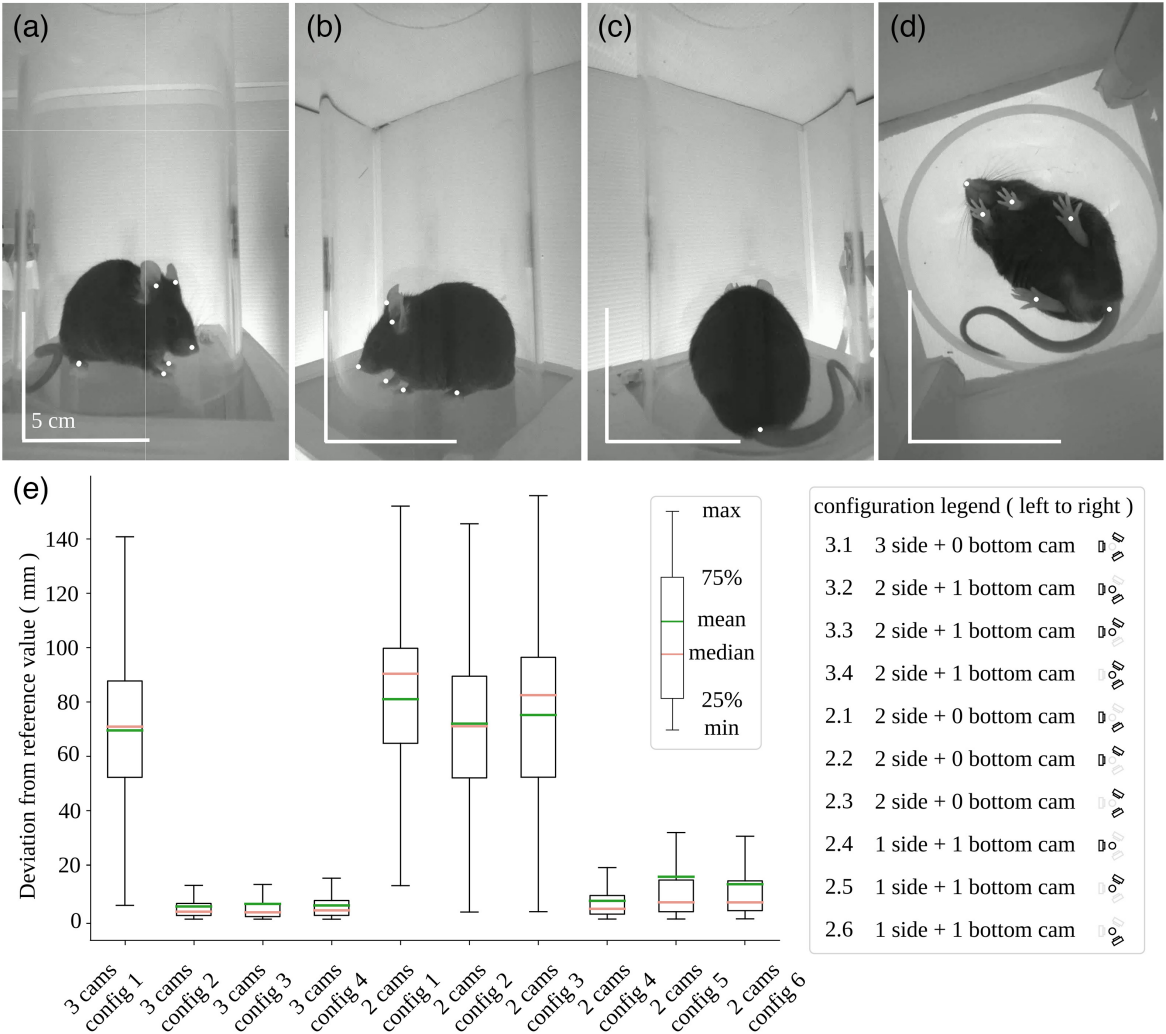
Multiview 89 mm mouse arena and 3D deviation from four-camera reference, reconstruction using single mouse subjects. (a)–(d) The frame of the source video captured from four different camera views. Keypoints are highlighted with white points. The 5 cm scale bar was estimated based on the diameter of the cylinder. It is varied for each camera view. (e) Boxplot of error relative to the reference positions; in this case, the four-camera predictions were taken to be the reference. The data used for these error estimates consist of video recording for a total of four different mice. Each video recording contained 1914 frames (64 s).

**Fig. 4:**
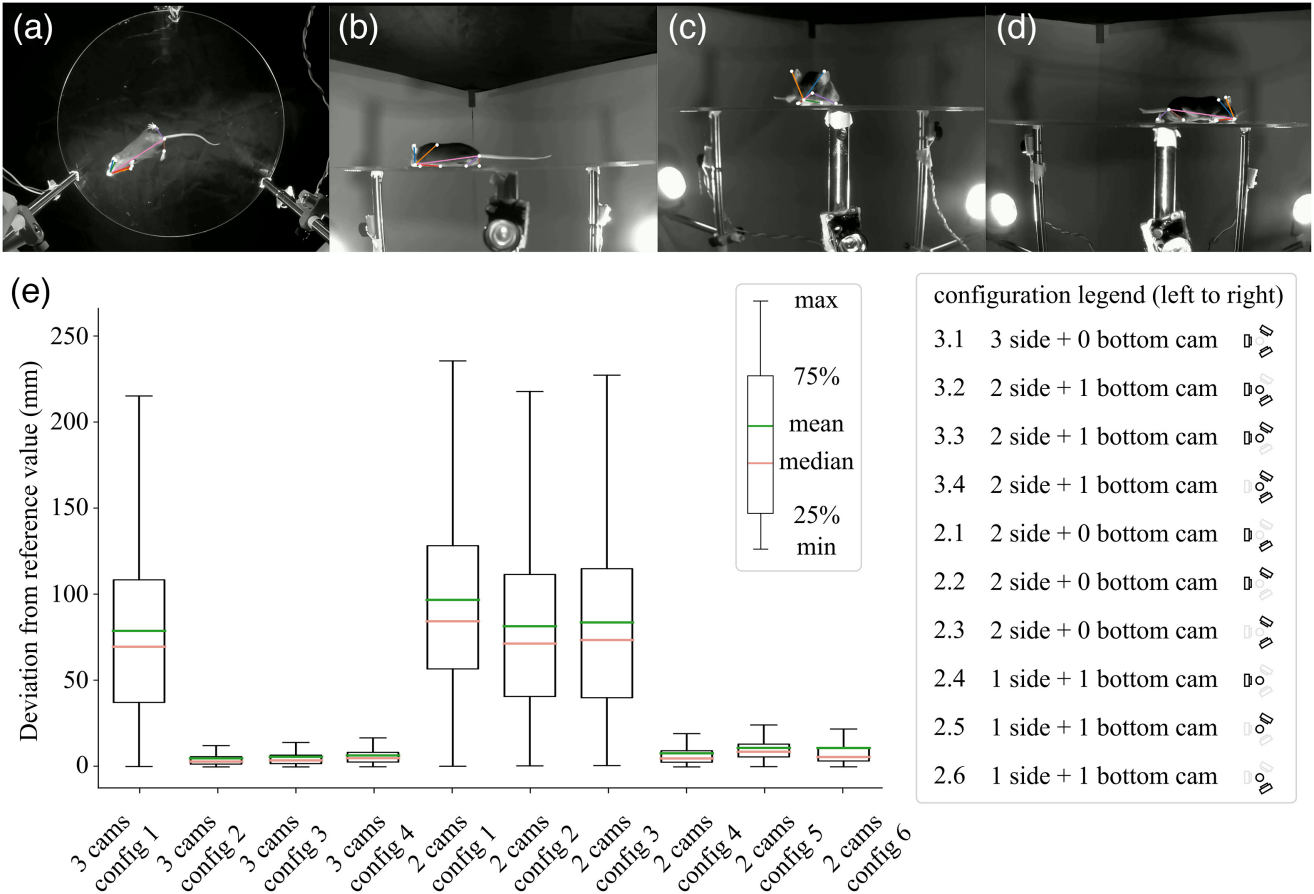
Multiview 320 mm mouse arena and 3D deviation from four-camera reference. Reconstruction using single mouse subjects. (a)–(d) The frame of the source video captured from four different camera views. Keypoints are highlighted with white points. (e) Boxplot of error relative to the reference positions; in this case, the four-camera predictions were taken to be the reference. The data used for these error estimates consist of video recording for a total of five different mice. Each video recording contained 9000 frames (300 s).

To determine the performance of the 3D reconstruction, we then employed a 3D-printed test object with known dimensions and unique features visible to multiple cameras [Fig. 1(e)]. The test object was attached to a thin rod with Blu-Tack reusable adhesive to minimize occlusion and presented and rotated in the capture volume. From the videos, the locations and trajectories of the keypoints were reconstructed using Anipose.^11^ Inter-keypoint distances were assumed to be valid proxies for the accuracy of the setup and reconstruction algorithm. Hence, the evaluation was conducted on the aggregated statistics of the distances between processed keypoints on the object. Results were then plotted as a deviation from a ground truth (Euclidean distance) and boxplots of error were constructed (Fig. 2). In the case of errors derived for actual mouse keypoints, four-camera Anipose coordinates were used as reference values in lieu of true ground truth (Figs. 3 and 4).

### 2.2 3D Test Object

The test objects are comprised of the letters X, Y, and Z each attached to a rod, positioned along the corresponding axis. The three rods converge at the origin such that all rods are perpendicular. The objects were modeled using Blender and fabricated using a Makerbot Replicator 3D printer. Diagrams of the test objects are included in Figs. 1(c)–1(f). The XYZ letters ensure enough asymmetry to provide unique visual cues for keypoint detection while remaining visually straightforward for intuitive use. This simple task gives rise to the high accuracy of 2D keypoint detection (the accuracy of Scene Text Recognition has reached over 98%^15^) compared to complex tasks such as body part detection.

### 2.3 Animal Behavior Data

As a proof of principle, behavioral data were acquired from surplus C57BL/6 mice of mixed genotypes (males, 3 to 8 months of age; n=4 for the 89 mm arena; n=5 for the 320 mm arena). Equal numbers of frames were used from each subject and data were analyzed in a concatenated manner. Potential errors in 2D keypoint detection were controlled by implementing the Viterbi filter in Anipose.^11^ While the Viterbi filter may help with animal pose determination, it may also introduce potential drifting artifacts across multiple frames when keypoints are placed on filtered feature locations. All procedures were conducted with approval from the University of British Columbia Animal Care Committee and in accordance with guidelines set forth by the Canadian Council for Animal Care. For this work images obtained from an Arducam quad camera system and 4 Picams were combined with little apparent difference in accuracy.

## 3 Results

The objective of our image acquisition and analysis pipeline is to provide a robust method of verifying 3D reconstruction sampling and accuracy for animal imaging arenas [Figs. 1(a) and 1(b)]. To accomplish this, it is assumed that the preservation of 3D-printed object inter-keypoint distance is a metric for reconstruction accuracy [Figs. 1(c) and 1(d)]. We also provide details on the construction of a standardized animal 3D pose capture arena and a software pipeline to evaluate the camera setup (see Sec. 2) for both small arena (89 mm diameter) and large arena (320 mm diameter). We have tested quad-camera configurations of monochrome synchronized Arducams or Raspberry Pi camera systems. In our experiments, the frame rate was set to 30 frames per second and four cameras were placed around the test objects or mice (Fig. 1).

Initial calibration of the arena was performed using ChArUco board (7×7, checker size: 10 mm, marker size: 8 mm, dictionary: AruCo DICT_4x4) as employed previously.^11^ 3D printed test objects were then utilized to check the quality of 3D reconstruction [Figs. 1(c) and 1(d)]. The objects have defined characteristics such as known distances between keypoints and asymmetries which provide unique object views on multiple cameras. We emphasize that the 3D printed test object is used to confirm accuracy but is not actively involved in calibration itself.

To assess the differences between 3D representations of the test object and its actual dimensions, Anipose^11^ was used to reconstruct the test object (from video images, without 2D filter) in 3D to calculate observed inter-keypoint distances (Euclidean distance). During the analysis, two types of systematic errors were observed for the test object: (1) bias that likely reflects the difference between the expected value and the true value (typically 1 to 2 mm for inter-keypoint distances) and (2) variance that refers to noise around repeated measures of the same keypoints.

The test object was also used to investigate the effect of camera number and camera position configuration and filter usage on the final prediction (Fig. 2). Given the presence of outliers, mean errors do not accurately reflect the broader distribution. Particularly within the larger arena (320 mm), the mean value could consistently surpass the maximum distance delineated by the boxplot [Figs. 2(a) and 2(c)]. Therefore, we suggest that readers focus on the distribution and the median. In general, the test object confirmed relatively accurate 3D sampling as the median error for small arena test object points was 1.21 mm for four camera configurations. In the larger arena, median error for test object points was significantly greater than the small arena [Figs. 2(a) and 2(c)], but the error still was small enough to report mouse body part locations (3.89 mm). Except for a few outliers, the error showed a relatively flat relationship with distance from the camera center (Spearman r=0.303, p value<0.01; based on four camera views), indicating consistent sampling across most of the field. However, the values outside of the arena border >486  pixels should be avoided for best performance (Fig. S1 in the Supplemental Material). In both arenas, the two-camera configurations exhibited higher median error and higher variance than three-camera configurations [Figs. 2(a) and 2(c)]. Specifically, the median errors in the smaller arena using the two- and three-camera configurations were 3.49 and 1.27 mm, respectively. Similarly, the median errors in the larger arena using the two- and three-camera configurations were 4.56 and 4.24 mm, respectively. This finding confirms that increasing the number of camera views can improve the accuracy and robustness of 3D reconstruction systems.^16^ Interestingly, the use of filters was observed to have no significant effect on either the bias and variance of the aggregate error for test 3D printed objects [Figs. 2(b) and 2(d)]. We caution that the lack of a filter effect may be related to the relatively slow smooth movement of the test object. It results in the camera frame rate significantly exceeding the motion frequency. Furthermore, due to the uncomplicated structure of the test object, DeepLabCut is capable of maintaining a remarkably high level of accuracy when the video quality reaches a sufficient level of quality. In this context, the application of a median filter typically exerts minimal influence on the data.

**Fig. 5:**
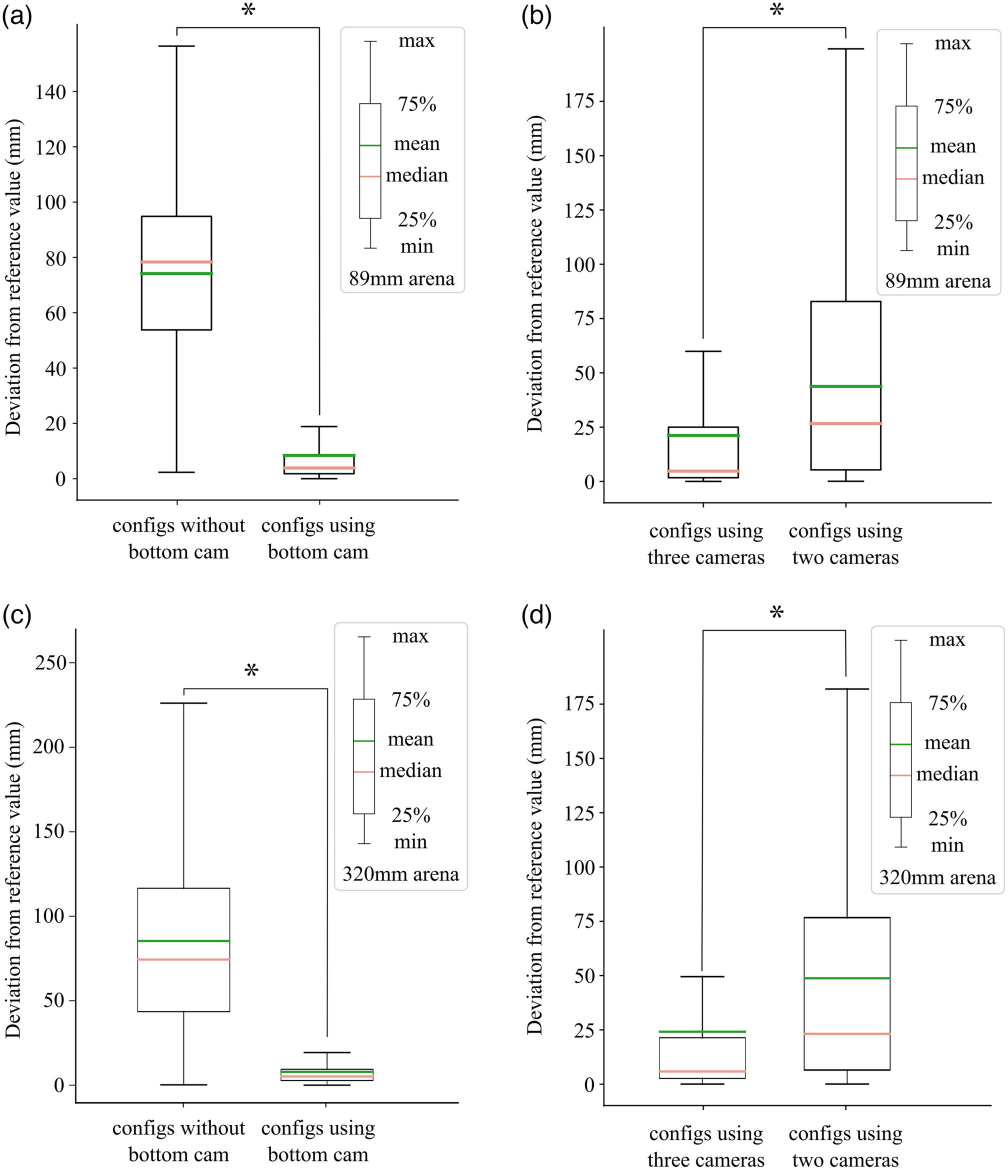
Multiview mouse arena and 3D deviation from four-camera reference reconstruction for mouse data accuracy and dependence on camera configuration and number. (a)–(d) Illustration of the effects of including the bottom camera and the number of cameras under different arena layouts (89 mm arena and 320 mm arena), respectively, on the boxplot of errors relative to the reference positions. The reference values and data are identical to those used in Fig. 3(e).

We evaluated videos of actual mice within the arena using the four-camera configuration [Figs. 3(a)–3(d), Figs. 4(a)–4(d)]. In this case, it was not possible to have a predefined ground-truth measurement for body part dimensions and position (as we had with the test object). Therefore, we employed results from the four-camera setup as a standard and calculated the deviation from this standard for the nine other camera configurations that used three or fewer cameras [Euclidean distance, Figs. 3(e) and 4(e)]. A two-way ANOVA was performed to analyze the effect of camera number/position configuration on the deviation from the prediction of four-camera standard. The significance of interaction between the effects of camera position configuration and camera number varied across arena size (F=0.08, p=0.78; 7656 frames pooled across mice in the smaller arena; F=723, p<0.001; 45,000 frames pooled across mice in the larger arena). In both arenas, Tukey post hoc comparisons showed that as expected lower camera number did significantly increase deviation from the four camera prediction [small arena: p=0.001, large arena: p=0.001, Figs. 5(b) and 5(d)]. Furthermore, camera position configuration also had a significant effect on error [small arena: p=0.001, large arena: p=0.001, Figs. 5(a) and 5(c)]. Errors for the mouse data were also relatively small (small arena: 3.83 mm, large arena: 5.74 mm) when the bottom camera and at least one side camera was present, but errors were quite large (small arena: 51.93 mm, large arena: 74.56 mm) when we removed the bottom sampling camera [Figs. 3(e) and 4(e)]. In general, we found that three-camera setups result in much smaller bias and deviations compared to two-camera setups (small arena: mean difference = 16.95 mm, p=0.001; large arena: mean difference = 23.36 mm, p=0.001; when all configurations were examined).

## 4 Discussion

For most scenarios where cameras are used in wild environment settings, it is impossible to ensure that arbitrarily placed cameras are calibrated. Here, we focus on the special conditions of laboratory animals in predefined arenas where cameras can be reproducibly placed and calibrated. 3D test specimens provide a means for 3D accuracy evaluation through the use of customizable and shareable 3D printed objects. 3D test objects work best within small animal motion capture studios with fixed number of camera and viewing angles allowing for confirmation of 3D relationships when recording. Such an approach has a potential advantage over existing 3D animal methods that determine accuracy and precision by projecting the results back into 2D to use measurements on a 2D plane as a proxy for 3D accuracy and precision.^11^ Small animal image capture approaches can constrain acquisition to compact-footprint camera studios where subjects can be viewed simultaneously from multiple perspectives. Although this approach requires the calibration of multiple cameras and verification of 3D reconstruction algorithms using test objects, this method can both reflect the challenges associated with 3D animal capture and serve as ground truth data.

By validating the performance on 3D test objects, the ability of a 3D tracking system to satisfy the constraints for rigid motion can be assessed. Skeletal driven animal movements can be treated as the combined motion from rigid body parts.^17^ To describe rigid body motion, two constraints must be satisfied: the preservation of length between arbitrary pairs of points within the rigid body parts during movement and the cross product between arbitrary pairs of vectors within the rigid body part.^18^ Therefore, 3D tracking systems must estimate both the length of bones in the skeleton and the relative angles of connecting joints (cross product) to accurately capture rigid body motion. For example, both jumping and walking are depicted by the rotation of bones rather than their elongation. In the 3D plane, both rotation and elongation may be visually similar in the 2D plane. The utility of the proposed 3D object method includes guiding hardware design, arena layout, error analysis, and benchmarking different reconstruction algorithms.

First, a main advantage of the proposed method over current tools is that 3D test objects can provide guidance on setups for multi-camera acquisition. Using a large number of cameras within an array is not only expensive but also can be challenging as solutions for multiple camera synchronization are often not readily available. Although an increase in camera count will yield better results (as reported in Sec. 3), a wise balance between cost and performance can and should be evaluated. Surprisingly, the increase in camera count in this study did not significantly improve the error measurements such as bias and variance in some cases. One possible explanation is that keypoints might already be accurately predicted when they can be robustly detected from two distinct camera views, rendering additional camera views superfluous. In these cases, keypoint error may be due to other factors than poor camera coverage. At the very minimum, we suggest that these multi-camera test objects can be used to assess a reasonable level of camera coverage for a particular acquisition arena when assessing mouse behavior. Second, the proposed method of 3D test objects helps analyze errors and parameters (or settings) for refinement. Excluding error related to camera placement, a residual amount of error was also observed and maybe associated with other factors such as neural network-based software for 2D pose estimation and the refinement based on temporal information from video recording. For example, we have also made the same error measurements on laboratory animals using four camera data as a reference point. By comparing the performance of the 3D tracking system, other settings can potentially be fine-tuned by taking advantage of test objects, such as frame rate, shutter speed, and type of filters. Hence, we suggest that users examine whether apparent levels of error are low enough to allow the use of a camera setup for hypothesis testing and thus minimizing the cost of the acquisition rig and fine-tuning the parameters based on the result from test objects. Third, 3D printed test objects can also provide an opportunity to benchmark different means of 3D pose estimation approaches. Prior studies often trained the neural network and refined their algorithm using different datasets. To assess the generalization of different algorithms, an unseen feature in testing data is required. Standard test objects could give rise to reliable benchmarks for different 3D pose estimation approaches. As none of the existing algorithms are trained with similar objects, we suggest it can serve as a reasonable method to evaluate the ability of different algorithms to generalize to new data. Video of the movement of ChArUco Checkerboards are typically used for 3D-calibration. However, the movement of these 2D boards needs to be done with care to ensure viewing by multiple cameras and that a 3D volume is sampled. We suggest that the 3D calibration objects we employ can be used as a means of confirming a proper 2D checkerboard calibration. The test object could also potentially be used as a means of calibration itself in a similar manner to the ChArUco board. Prior studies already implemented an L-frame to do the extrinsic camera calibration instead of Checker/ChArUco board.^5^

However, several questions remain unanswered at present. One could question how well the XYZ test object can be applied to a specimen, such as a mouse, and whether it has a similar complexity as a 3D printed structure. Ideally, one would desire objects to be captured by multiple cameras without occlusion. Because the test object represents such a near-optimal case, the test object can only provide the lower bound of 3D reconstruction error from multiple cameras. Any occlusion might result in the deterioration of 3D tracking system performance. Thus, a bit of redundancy (overlapping camera coverage) in the 3D recording system is suggested. In the case of the mouse data, frequent occurrences of occlusion from the side perspective significantly compromised accuracy. However, when employing the test object, the advantages of the bottom view over the side view were not distinctly apparent. This result may be explained by the fact that semantic keypoints (paws, limbs, etc.) of mice tend to have more details visible from the bottom view than the sides. Therefore, while the use of test objects can provide the lower bounds of expected errors, one should always optimize views based on keypoints of importance within the object that is being tracked.

## 5 Conclusion

Overall, our study suggests that the 3D printable test objects and standardized studios help constrain acquisition parameters offering neuroscientists the ability to focus on developing unique behavioral paradigms.

## 6 Appendix: Supplementary Material

Four videos and a figure are included as supplemental material:

Video 1. Video of 3D test object 3D keypoint predictions in 89 mm arena (MP4, 11.0 MB [URL: https://doi.org/10.1117/1.NPh.10.4.046602.s1]).
Video 2. Video of animals’ 3D keypoint predictions in 89 mm arena (MP4, 11.7 MB [URL: https://doi.org/10.1117/1.NPh.10.4.046602.s2]).
Video 3. Video of 3D test object 3D keypoint predictions in 320 mm arena (MP4, 11.5 MB [URL: https://doi.org/10.1117/1.NPh.10.4.046602.s3]).
Video 4. Video of animals’ 3D keypoint predictions in 320 mm arena (MP4, 20.9 MB [URL: https://doi.org/10.1117/1.NPh.10.4.046602.s4]).

(Fig. S1 in the Supplemental Material). Illustration of the effects of distance to image center on inter-keypoint error. (320 mm arena).

## Code, Data, and Materials Availability

The source codes are available for downloading at the link in Ref. 19.
