# Supplementary figures and images for: Standardized 3D test object for multi-camera calibration during animal pose capture
Source: Neurophotonics. 2023 Nov 7;10(4):046602. doi: 10.1117/1.NPh.10.4.046602 (PMC10629347; doi:10.1117/1.NPh.10.4.046602)

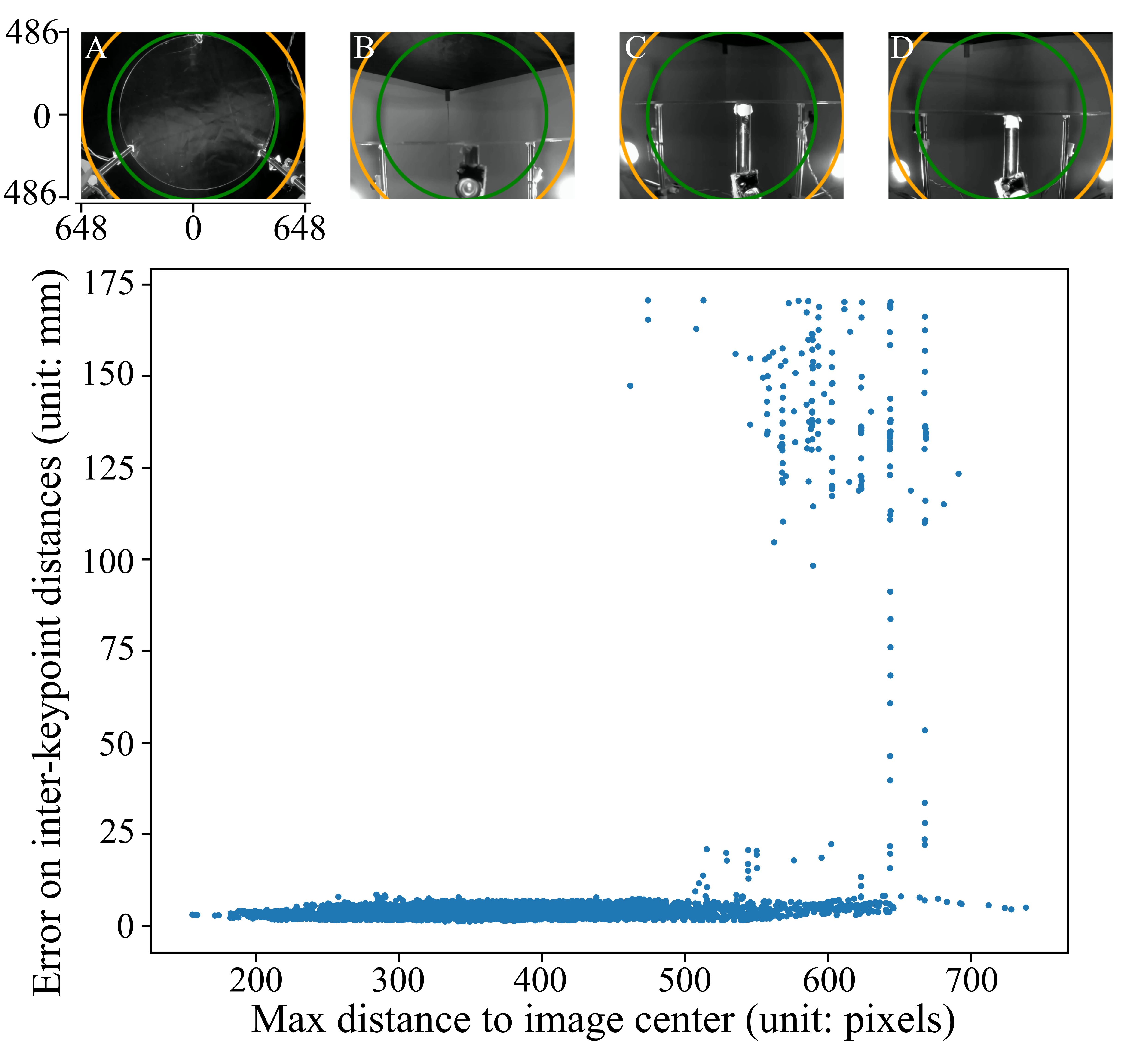

Supplement: Supplementary file 1 [file NPh_010_046602_SD001.png]
